# Fifty years of research on questionable research practises in science: quantitative analysis of co-citation patterns

**DOI:** 10.1098/rsos.230677

**Published:** 2023-10-18

**Authors:** Michelle Jin Yee Neoh, Alessandro Carollo, Albert Lee, Gianluca Esposito

**Affiliations:** ^1^ Psychology Program, School of Social Sciences, Nanyang Technological University, Singapore 639818, Singapore; ^2^ Department of Psychology and Cognitive Science, University of Trento, Rovereto 38068, Italy

**Keywords:** questionable research practises, scientific integrity, ethics of research

## Abstract

Questionable research practises (QRPs) have been the focus of the scientific community amid greater scrutiny and evidence highlighting issues with replicability across many fields of science. To capture the most impactful publications and the main thematic domains in the literature on QRPs, this study uses a document co-citation analysis. The analysis was conducted on a sample of 341 documents that covered the past 50 years of research in QRPs. Nine major thematic clusters emerged. Statistical reporting and statistical power emerged as key areas of research, where systemic-level factors in how research is conducted are consistently raised as the precipitating factors for QRPs. There is also an encouraging shift in the focus of research into open science practises designed to address engagement in QRPs. Such a shift is indicative of the growing momentum of the open science movement, and more research can be conducted on how these practises are employed on the ground and how their uptake by researchers can be further promoted. However, the results suggest that, while pre-registration and registered reports receive the most research interest, less attention has been paid to other open science practises (e.g. data sharing).

## 1. Introduction

Although scientific misconduct such as fraud, data falsification and plagiarism are more widely covered in mainstream media, concerns regarding the credibility of research are also linked to questionable research practises (QRPs). QRPs have been defined as ‘design, analytic or reporting practices that have been questioned because of the potential for the practice to be employed with the purpose of presenting biased evidence in favour of an assertion’ [1, p. 3]. Examples of such QRPs include selective reporting of dependent measures in a study, decisions on collecting more data after checking the significance of results, and rounding off a *p*-value. An article by John *et al.* [2] reported high estimates of the prevalence of QRPs, with some estimates approaching 100%, which the authors proposed to be suggestive of these practises as a *de facto* norm. Their article garnered widespread attention and threw a spotlight on the prevalence of these QRPs in scientific research. Publication pressure has been cited as one major factor for the prevalence of QRPs in science [3]. The prevalence of QRPs has drawn into question the quality of science, where the selective reporting and publication of results biases effect sizes upwards [4]. This publication bias, where reporting and publication tend to be biased towards findings of statistical significance, can also be attributed to publication pressure as well as the low likelihood of journal publication of null or negative results, where null findings were only reported in 4% of studies in a review of education and school counselling psychology journals [5]. For example, strong results were both more likely to be published as well as written up by authors compared to null findings [6]. Notably, Simmons *et al.* [7] reported how QRPs such as flexibility in choosing among dependent variables and sample size can contribute to the relative ease in deriving ‘false-positive’ results through a series of simulations and experiments. Such QRPs raise concerns about the credibility of research findings, including the impact of QRPs on the replicability of published scientific results. Hence, QRPs have also been cited as a contributing factor to the replication crisis in science, which refers to the inability to replicate findings across many areas of research [8,9].

This study aimed to identify the key publications and trends in research conducted on QRPs, including the focus of these publications and gaps in the literature, in order to generate a clearer picture of the status and characteristics of research into QRPs in science. Although a number of reviews have been conducted on QRPs, most of them tend to be conducted with a scope that is focused within specific fields of science—for example, the review conducted by Banks *et al.* [10] concentrated on the social sciences. Moreover, reviews appear to be focused on estimating the prevalence of different types of QRPs (e.g. [11]). Research adopting a broader perspective in examining research on QRPs in science as a whole is still very limited as pointed out by the review by Aubert *et al.* [12] about research integrity. In comparison to systematic reviews and meta-analyses which usually address one research topic in scope, a scientometric approach can provide a more holistic view of the multiple perspectives to the debate and research conducted on QRPs. In addition, the scientometric results also indicate the temporal shifts in these research trends.

To our knowledge, a scientometric perspective has not been previously applied to QRP research as a whole. A scientometric review can take into account a significant majority of the literature on QRPs across different scientific fields and provide insight into key research topics and articles in the field as well as their relationships using a data-driven approach. The scientometric perspective allows the identification of research trends and the direction of research into QRPs in science as a whole across time. To this end, references and relevance of publications in the existing literature were analysed with a document co-citation analysis (DCA) [13–15], which allows for the modelling of quantitative relationships between a large sample of documents and their citations.

## 2. Material and methods

In line with the standardized and established procedures [14,16], publications were downloaded from Scopus using the following search string TITLE-ABS-KEY (‘questionable research practice*’). Titles and abstracts of the retrieved documents were checked manually to ensure we were collecting the literature of interest with minimal noise from non-relevant publications. This string term was used so as to direct univocally to the literature of interest with minimal noise, as opposed to specifying particular QRPs which may skew the search output in that particular direction rather than providing a more comprehensive output. We chose to adopt this more conservative approach in the initial stage knowing that all the citations included in these selected papers would have been included in the analysis in the second stage. For this reason, relevant documents that did not precisely contain the keywords ‘questionable research practices’ in their title, abstract and keywords were still included in the study when they were cited by the initial sample of seed documents. Because Scopus covers a greater number of indexed journals and recent documents, it was selected to be the database. Only one database was used for the article search in order to have a clean database, as is the common practise in scientometric studies [17–19]. The search conducted on 6 February 2023 yielded 341 documents published from 1974 to 2023. The initial sample of documents was analysed with the *bibliometrix* package for R [20]. By doing so, the main information about the collected sample and the main co-occurrence of documents’ keywords were retrieved.

### 2.1. Data import in CiteSpace

Documents downloaded from Scopus were imported into CiteSpace software (v. 6.1.R6 64-bit Advanced) [21], which was used to conduct the scientometric analysis. If actual data loss was higher than the expected one (1–5% of the total references), references were manually corrected. An amount of 19 480 references cited by the documents were valid, out of a total of 19 863 (98.07%). A valid reference contains the following seven key pieces of information: author, year of publication, title, source, volume, pages and DOI [14,22]. Irregularities in the citation format resulted in a number of entries being considered invalid. The data loss of the current study represents a negligible data loss for scientometric reviews. Repeated entries were removed by using the CiteSpace function (Remove Alias), which was turned on [23].

### 2.2. Document co-citation analysis and parameter optimization

Main research domains were determined through the use of a DCA, which is based on graph theory principles. The basis of the DCA is the frequency with which two or more documents are cited together in source articles [24]. In DCA, frequent co-citations among documents are assumed to be reflective of clusters of research with a common research theme [15,25]. The resulting network from the DCA is made up of documents frequently cited together with the documents that cite them (i.e. articles downloaded from Scopus). In the network, single-cited documents are modelled as nodes while co-citations occurrences are modelled as links. In the network, the assignment of edge weights is according to the frequency of co-citations between documents.

A balanced network was obtained through optimization of DCA parameters. Several DCAs were computed and compared, each with a different setting for one of three node selection criteria; g-index, TOP *N*, TOP *N*%, as done in [26–28]. The node selection criteria referred to *a priori* settings which defined the selection criteria used for choosing articles to be included in the network, ultimately determining the generation of the final network. The g-index is a measure of the citation scores of an author’s top publications [29,30], where its value represents the largest number equalling the average number of citations of the most highly cited g publications [31]. TOP *N* and TOP *N*% are criteria used to select *N* and *N*% most cited within a time slice, which was set to 1 year in this study, as network nodes, respectively [14].

The final optimal network was generated through the computation of multiple DCAs with variations in node selection criteria and their scale factor values, which refer to the chosen numeric values used as thresholds for the respective node selection criteria [32]. Specifically, DCAs with the following node selection criteria were compared: g-index with scale factor *k* set at 10, 15, 20 and 25, TOP *N* with scale factor *N* set at 50, and TOP *N*% with scale factor *N* set at 10. By comparing the overall effects on the structural metrics of the generated network, the number of nodes included, and clusters identified, the node selection criteria and scale factor to use for the generation of the final network were determined. A DCA with g-index with the scale factor *k* set at 15 was the parameter used to generate the final network of articles.

**Figure 1.**
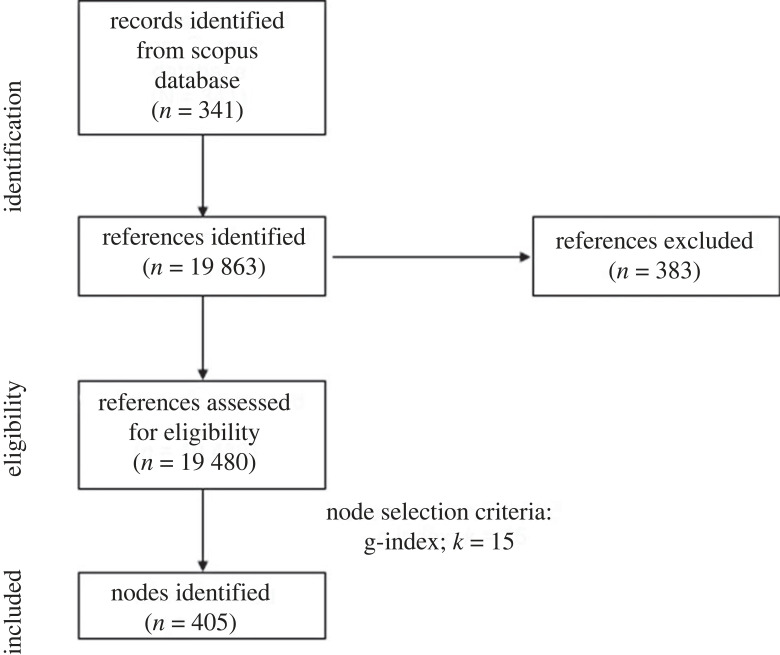
Preferred reporting items for systematic reviews and meta-analyses (PRISMA) flowchart for literature search and reference eligibility.

The literature search and the generation of the DCA network are summarized in figure 1.

### 2.3. Metrics

The CiteSpace results are described using structural and temporal metrics. Structural metrics include (i) *modularity-Q*, (ii) *silhouette scores,* and (iii) *betweenness centrality*. Modularity-Q values range from 0 to 1 and is indicative of the degree of the divisibility of the network into single groups of nodes, referred to as modules or clusters [33]. High modularity-Q values reflect a well-structured network [15]. The homogeneity of single clusters is measured using silhouette scores. Silhouette provides information on the clusters’ internal cohesion and separation from other clusters [34]. Values of silhouette ranges from −1 to +1. Higher silhouette score values represent greater separation from other modules and internal consistency [35]. Betweenness centrality represents the degree to which a node connects an arbitrary pair of nodes in the network [14,36]. Betweenness centrality values range from 0 to 1, where groundbreaking and revolutionary works in the scientific literature usually had higher scores [33].

Temporal metrics consist of (i) *citation burstness* and (ii) *sigma*. The Kleinberg’s algorithm [37] is used to calculate citation burstness, which indicates a sudden increase in the number of citations of an article within a given time frame [38]. Citation burstness provides information on the impactful documents within the network. Sigma is calculated with the equation (centrality + 1)^burstness^ and indicates a document’s novelty and its influence on the overall network [39].

Modularity-Q and silhouette scores were used to examine the overall configuration of the generated network and identified clusters of references. Betweenness centrality and temporal metrics were used to examine the attributes of single nodes in the network.

### 2.4. Narrative review

In the Discussion section of the current work, the identified clusters will be reviewed in greater detail following a narrative approach. In this way, the current work aims to integrate the insight from the quantitative analysis of the scientometric approach to the qualitative insight from a narrative review. To review the clusters, first, the documents that cite the most number of references found in the cluster (i.e. the major citing articles) are identified. These major citing documents contribute to a significant number of links in the network and in the clusters. The major citing documents are then analysed for their research focus and the common theme(s) between them, which are then discussed in relation to the cited articles in the cluster. Identifying common themes and links between the citing articles and cited articles can provide insight into deriving the main theme of the cluster, and highlight the key research topics, arguments and significant findings of the cluster.

## 3. Results

### 3.1. Bibliometric analysis on the citing documents

The sample of documents downloaded from Scopus expanded from 1974 to 2023 with an annual growth rate of 4.89%. On average, each document obtained 29.34 citations with an average document citation by year of 4.032. The most frequently cited documents were authored by John *et al.* [2] (total citations = 1130; total citations per year = 94.2) and by Fanelli [40] (total citations = 968; total citations per year = 64.5).

**Figure 2.**
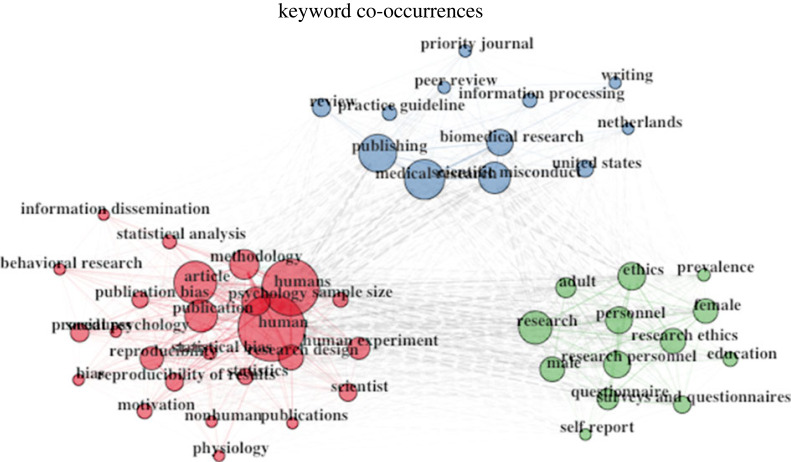
Top 50 keywords co-occurrences.

A total of 659 keywords selected by the authors indexed the documents. The most popular keywords plus were *questionable research practices* (n=121 documents), *research integrity* (n=33 documents), *open science* (n=30 documents), *research misconduct* (n=29 documents), *publication bias* (n=26 documents), *replication* (n=25 documents), *reproducibility* (n=20 documents), *research ethics* (n=19 documents), *replicability* (n=18 documents) and *meta-analysis* (n=15 documents; see figure 2).

In the citing documents, a total of 870 unique authors were identified. On average, the data sample included 0.392 documents per author and an average of 3.17 co-authors per document. In 26.98% of cases, documents were published with international collaborations.

The results indicated that the five most productive authors were Wicherts JM (10 published documents), Bouter LM (nine published documents), Banks GC, Brown M and Sacco DF (all with seven published documents). Authors’ affiliations were mostly from the USA (n=80 documents; frequency = 0.3150; single country publications (SCP) = 61; multiple country publications (MCP) = 19), The Netherlands (n=47 documents; frequency = 0.1850; SCP = 33; MCP = 14) or from the UK (n=22 documents; frequency = 0.0866; SCP = 14; MCP = 8).

Finally, the main sources on documents regarding QRPs emerged as *Science and Engineering Ethics* (n=17 documents), *PLoS ONE* (n=15 documents), and the *Journal of Empirical Research on Human Research Ethics* (n=12 documents).

### 3.2. Structural metrics

The final optimized network obtained from the DCA consisted of 405 nodes (i.e. documents) with 1499 links (i.e. co-citations), which indicates an average of 0.27 connections with other references for each node. The network had a modularity-Q index of 0.747 and a mean silhouette score of 0.904, indicating high divisibility of the network into homogeneous clusters (i.e. thematic domains of research).

### 3.3. Thematic clusters

**Figure 3.**
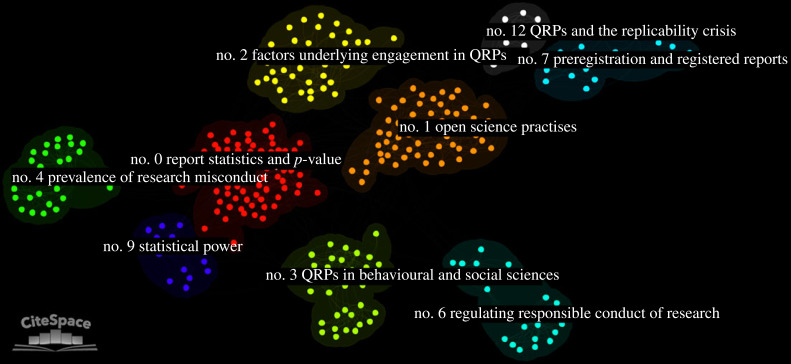
Document co-citation analysis network of all literature on questionable research practises (QRPs). The network is created using graph theory principles, which allows the representation of the relationship between items through the use of edges and nodes. The graph represents the existing relationships in the literature on QRPs by using documents as nodes and co-citation patterns as edges. The nine major clusters are grouped by colour. The image was generated with the CiteSpace software [21].

From the analysis of the relationships between documents in the final network, a total of nine major clusters were identified (figure 3 and table 1). Based on the log-likelihood ratio (LLR) algorithm, each cluster was initially labelled with a label automatically generated by the algorithm. A manual visual inspection was then conducted to propose more suitable labels where necessary in order to better reflect the thematic interest of the documents in the cluster. The largest cluster no. 0 consisted of 82 nodes and had a silhouette score of 0.848, with the constituent references being published in 2012 on average. The cluster was manually labelled ‘reporting statistics and *p*-values’. Second, cluster no. 1 consisted of 60 nodes and had a silhouette score of 0.856, with the constituent references being published in 2015 on average. The cluster was manually labelled ‘open science practises’. Third, cluster no. 3 consisted of 35 nodes and had a silhouette score of 0.916, with the constituent references being published in 2011 on average. The cluster was manually labelled ‘QRPs in behavioural and social sciences’. Cluster nos 4 and 6 had the oldest mean year of publication (2005 and 2006, respectively), whereas cluster nos 2 and 7 were the most recent clusters with a mean year of publication in 2016. Based on their silhouette score, each major cluster results to be highly homogeneous and well separated from the others.

**Table 1.** Metrics of the nine clusters identified with the document co-citation analysis. (For each cluster, the number of included documents (i.e. size), the silhouette score, its mean publication year, the log-likelihood ratio (LLR) label and the suggested label are presented. Silhouette score is a metric that provides information on the degree of a cluster’s homogeneity and separation from other clusters. LLR labels are automatically generated by the software.)

cluster ID	size	silhouette	mean publication year	LLR label	suggested label
0	82	0.848	2012	questionable research practise	reporting statistics and *p*-value
1	60	0.856	2015	open science era	open science practises
2	40	0.895	2016	questionable research practise	factors underlying engagement in QRPs
3	35	0.916	2011	guest commentary	QRPs in behavioural and social sciences
4	32	0.998	2005	research	prevalence of research misconduct
6	24	0.977	2006	responsible conduct	regulating responsible conduct of research
7	19	0.950	2016	registered report	preregistration and registered reports
9	15	0.972	2010	ironic effect	statistical power
12	9	0.996	2012	past	QRPs and the replicability crisis

### 3.4. Citation burstness

A total of five documents exhibited a citation burst (table 2). Considering that these documents had a period characterized by an abrupt increase in the number of received citations, we considered these documents as the most influential in the network. Three of these documents belonged to cluster no. 0, one to cluster no. 3, and one to cluster no. 7. The article with the strongest and longest citation burst was authored by Simmons *et al.* [7] with a score of 6.63, with the burst beginning in 2014 and ending in 2019. The same article also had the highest sigma value of 2.13. The publication by the Open Science Collaboration [41] has a burst ending in 2023, which probably indicates that the document is still having an influence on the overall literature.

**Table 2.** Five publications with a citation burst. (Citation burstness was defined as an abrupt increase in the number of citations received by a document within a period of time [14,36].)

reference	burst	publication year	burst begin	burst end	duration	centrality	sigma
Simmons *et al.* [7]	6.63	2011	2014	2019	5	0.12	2.13
Open Science Collaboration [41]	4.69	2015	2021	2023	2	0.03	1.16
Button *et al.* [42]	4.32	2013	2014	2017	3	0.03	1.11
Simonsohn *et al.* [43]	4.25	2014	2015	2017	2	0.01	1.05
Fanelli [40]	4.08	2009	2016	2017	1	0.02	1.07

## 4. Discussion

Each cluster will be discussed in terms of the citing articles and cited references in order of recency; from the oldest to most recent average year of publication of the cluster. The main citing articles in each cluster will be highlighted and their coverage and global citing score (GCS) will be reported. Coverage refers to the number of articles in the cluster that were cited by the citing article and GCS refers to the total number of citations received by a paper as indexed on Scopus.

### 4.1. Cluster no. 4: prevalence of research misconduct

The major citing articles in cluster no. 4 were authored by Katavic [44] with a coverage of 16 articles and GCS of 6 and Fanelli [40] with a coverage of 16 articles and GCS of 968. This cluster appears to be the initial body of work focusing on research into the prevalence of research misconduct in the scientific community, namely falsification, fabrication and plagiarism. Since the automatically generated LLR cluster label is ‘research’, the cluster label was manually replaced with ‘prevalence of research misconduct’ for greater clarity and specificity.

Both the citing articles [40,44] discuss the prevalence of research misconduct, where Fanelli [40] conducted a meta-analysis indicated a pooled weighted average of 1.97% of scientists admitting to the fabrication, falsification and modification of data or results at least once. Similarly, the cited references investigate the prevalence of research misconduct, including falsification and fabrication, and conflicts of interest [45–48]. Given the relatively early mean publication year of the cluster (2006), this cluster appears to be the beginning of research into the prevalence of research misconduct and integrity in the wake of several high-profile cases of scientific fraud such as fake stem cell lines [49]. The cluster suggests that research into QRPs began as research into more ‘overt’ and ‘serious’ cases of research misconduct such as falsification or fabrication of data and results, and conflicts of interest, before research began to focus on investigations into QRPs.

### 4.2. Cluster no. 6: regulating responsible conduct of research

The major citing articles in cluster no. 6 were authored by Wester *et al.* [50] with a coverage of 16 articles and GCS of 10, DiLorenzo *et al.* [51] with a coverage of five articles and GCS of 1, and Pupovac *et al.* [52] with a coverage of three articles and GCS of 14. The focus of the articles in cluster no. 6 appear to be related to the definition and regulation of research misconduct. Since the automatically generated LLR cluster label is ‘responsible conduct’, the cluster label was manually replaced with ‘regulating responsible conduct of research’ for greater clarity and specificity.

In relation to definitions, a number of articles discuss the definitions for what constitutes as research misconduct or QRPs, and how more precision and clarity in these definitions can help to foster integrity in research [52–54]. For example, Pupovac *et al.* [52] proposed that a lack of formal documentation instituting research integrity and clarifying forms of research misconduct and QRPs in the University of Rijeka in Croatia may be one contributing factor to the higher rates of research misconduct observed in their study. In line with the need for greater clarity and enforcement of research integrity in institutions, there is also a need for the education of researchers of these definitions of research integrity and research misconduct. Hence, the cluster also includes investigations and discussions of education pertaining to research integrity for researchers and its role in preventing research misconduct, where it has been proposed to be critical [55]. Pupovac *et al.* [52] highlighted a lack of formal education about responsible conduct of research for Croatian scientists. Similarly, DiLorenzo *et al.* [51] also proposed that more formal considerations of education and training in responsible conduct of research may be beneficial. Finally, in line with the notion of greater clarity in the definition of research misconduct and QRPs, there are also articles in this cluster that relate to data collection for self-reports of such behaviour. For example, the major citing article by Wester *et al.* [50] is a pilot study for the development of a self-report measure (responsible conduct of research measure) to examine research misconduct. Similarly, there is also a cited reference for the Scientific Misconduct Questionnaire-revised [56].

### 4.3. Cluster no. 9: statistical power

The major citing articles in cluster no. 9 were authored by Schimmack [57] with a coverage of 13 articles and GCS of 280, Bakker *et al.* [58] with a coverage of 11 articles and GCS of 462. The main theme of this cluster are statistical power concerns in studies, specifically, the relationship between QRPs and underpowered studies. Since the automatically generated LLR cluster label is ‘ironic effect’, the cluster label was manually replaced with ‘statistical power’ for greater clarity and accuracy.

Both major citing articles highlight the inflation of effect sizes and high rates of false positive results when using several small studies with insufficient power. Specifically, Schimmack [57] argues that QRPs may be a contributor to the reporting of too many positive results, coupled with lower statistical power in multiple-study articles since there is a greater number of statistical tests being performed. In order to avoid the QRPs of hypothesizing after the results (HARKing), it has been noted that more complex studies involving multiple hypotheses are being designed [59], possibly contributing to multiple-study designs. However, multiple-study articles in experimental psychology have low power, where it is extremely unlikely that a single series of studies yields only positive results, with Schimmack [57] suggesting a possible intensification in the pressure to engage in QRPs, further compromising the credibility of the research. Similarly, the cited references also discuss issues of statistical power in multiple-study experimental design [59] and the persistence of underpowered studies in psychology [60].

### 4.4. Cluster no. 3: QRPs in behavioural and social sciences

The major citing articles in cluster no. 3 were authored by Fanelli & Ioannidis [61] with a coverage of 15 articles and GCS of 100, Banks *et al.* [10] with a coverage of 12 articles and GCS of 77, and Banks *et al.* [1] with a coverage of 10 articles and GCS of 89. From the citing articles, the scope of the articles in this cluster appears to be the prevalence of QRPs in behavioural and social sciences such as psychiatry [61], psychology [62,63] and management [1]. Similarly, the scope of the cited references is also largely centred around these areas of study [64–68]. Since the automatically generated LLR cluster label is ‘guest commentary’, the cluster label was manually replaced with ‘QRPs in behavioural and social sciences’ to reflect the cluster content more accurately.

The nature of the method and measurements have been argued to influence the risk of bias, especially in fields of sciences where there is greater difficulty in replication, less clarity in theories and less standardization of methods [61]. Fanelli & Ioannidis [61] found that studies with behavioural measures tended to be more likely to report extreme effects. Similarly, the cited reference by Fanelli [69] reported increasing proportions of studies reporting positive results from physical to medical and social sciences. This cluster seems to suggest that there is a greater concern regarding the prevalence of QRPs in particular fields of science, given differences in methods and measures.

### 4.5. Cluster no. 0: reporting statistics and *p*-value

The major citing articles in cluster no. 0 were authored by Spellman [70] with a coverage of 12 articles and GCS of 106, Carter *et al.* [71] with a coverage of 12 articles and GCS of 202, and de Winter & Dodou [72] with a coverage of 11 articles and GCS of 35. The main theme in cluster no. 0 centres around QRPs relating to statistics and *p*-values and the implications on scientific reporting. Similarly, the cited articles in the cluster revolved around statistics [42,73,74], *p*-values [43,75] and positive results in science [7,69,76]. Since the automatically generated LLR cluster label is ‘questionable research practice’, the cluster label was manually replaced with ‘reporting statistics and *p*-value’ to reflect the cluster content more accurately and specifically.

As highlighted by Spellman [70], there have been longstanding concerns with the employment of null hypothesis significance testing and guidelines regarding the reporting of statistics in publications. Notably, *p*-values just below the commonly used threshold of 0.05 have been reported to be more prevalent, in part owing to selective publication of positive results [6] but in part, also attributable to false positive results. de Winter & Dodou [72] demonstrate an increase in *p*-values from 0.041 to 0.049, and argued that an increase in QRPs and structured reporting may be the cause for these longitudinal trends. QRPs related to these concerns pertaining to statistics reporting include *p*-hacking, where various statistical analyses are conducted on the data before positive results are selectively reported, and incorrect *p*-value rounding down, which can cause false positive results [2,7]. A number of articles in the cluster also discuss statistical methods to address these QRPs and publication bias. Simonsohn *et al.* [43,75] proposed the use of the *p*-curve—the distribution of *p*-values that are statistically significant for a set of studies, where they argued that right skewed *p*-curves are generated only by true effects. In addition, the major citing article by Carter *et al.* [71] reported that no single meta-analytic method showed better performance through a series of simulation studies including levels of QRPs, publication bias and heterogeneity. Hence, their recommendation was a move towards sensitivity analyses similar to what they have done, and conducting large-scale, preregistered replications.

### 4.6. Cluster no. 12: QRPs and the replicability crisis

The major citing articles in cluster no. 12 were authored by Swiatkowski *et al.* [77] with a coverage of nine articles and GCS of 64, and Stürmer *et al.* [78] with a coverage of five articles and GCS of 10. The issue of replicability in social psychology appears to be the main focus of this cluster, where QRPs are highlighted as one contributor to the replication crisis. It appears to be an initial body of work discussing systemic-level factors contributing to the low replicability of psychology results in the field. Since the automatically generated LLR cluster label is ‘past’, the cluster label was manually replaced with ‘QRPs and the replicability crisis’ for greater accuracy and clarity.

One such factor contributing to low replicability in psychology are QRPs, where the results from [78] indicated the prevalence of such QRPs could be primarily attributed to academic incentive structures. As highlighted in the work of cluster no. 0, QRPs tend to cause inflation of false positive results, leading to the low replicability of results. Moreover, it is concerning that [78] reported a moderate to high prevalence of such QRPs relating to statistical significance, underpowering and selective reporting. In line with the theme of this cluster, a number of replication studies are also cited [79,80], including the landmark study by the Open Science Collaboration [41].

### 4.7. Cluster no. 1: open science practises

The major citing articles in cluster no. 1 were authored by Agnoli & Carollo [81] with a coverage of 10 articles and GCS of 6, Patall [82] with a coverage of nine articles and GCS of 13, and Latan *et al.* [83] with a coverage of eight articles and GCS of 1. The main theme of the cluster appears to be the movement towards open science and open science practises, which have been proposed to address the prevalence of QRPs in science. Since the automatically generated LLR cluster label is ‘open science era’, the cluster label was manually replaced with ‘open science practises’ to reflect the cluster content regarding open science practices more specifically, as opposed to referencing a timeframe as suggested by the LLR label.

A number of citing articles discuss the movement towards open science and the implementation of open science practises in science [84–86]. For example, in the major citing article by Patall [82], open science strategies such as pre-registration and data/material sharing in the educational psychology field are discussed. Similarly, in the major citing article by Latan *et al.* [83], the authors reported a high prevalence of QRPs among business scholars, citing reasons such as publication pressure and manuscript rejection. The article also outlines recommendations including pre-registration and making research materials publicly available [83]. Essentially, transparency in the research process is the underlying principle of adopting open science practises in addressing QRPs [87], which is consistently highlighted by the articles in this cluster.

### 4.8. Cluster no. 2: factors underlying engagement in QRPs

The major citing articles in cluster no. 2 were authored by Damen *et al.* [88] with a coverage of eight articles and GCS of 0, Bruton *et al.* [89] with a coverage of seven articles and GCS of 7, and Maggio *et al.* [3] with a coverage of four articles and GCS of 17. The articles in this cluster focus mainly on investigating the factors that contribute to the engagement of QRPs in the scientific community. Since the automatically generated LLR cluster label is ‘questionable research practise’, the cluster label was manually replaced with ‘factors underlying engagement in QRPs’ for greater specificity and clarity.

These factors include researcher characteristics such as attitudes and opinions towards QRPs [90], personal motivations [91] and perceptions of publication pressure [92]. Similarly, there are cited articles investigating the basis for engagement in QRPs [93–95]. For example, the major citing article by Maggio *et al.* [3] reported several researcher characteristics such as age, publication numbers and geographical location. Moreover, perceptions of publication pressure was found to be the strongest individual predictor for scientific misconduct in their study. The results from studies in this cluster can be informative for initiatives targeting the use of QRPs in the scientific community such as interventions described in the citing articles (e.g. [89,96] and the cited reference by Kidwell *et al.* [97]) and promote greater transparency and adoption of the open science practises outlined in cluster no. 1. For example, Sacco *et al.* [90] reported that a 1 h training session for psychology graduate students resulted in attitude changes towards QRPs where the students found it less ethically defensible.

### 4.9. Cluster no. 7: preregistration and registered reports

The major citing articles in cluster no. 7 were authored by Noret *et al.* [98] with a coverage of five articles and GCS of 1, Cook *et al.* [99] with a coverage of four articles and GCS of 3, and Götz *et al.* [100] with a coverage of four articles and GCS of 88. This cluster appears to be a more niche body of work investigating the application of preregistration and registered reports in various research fields. Since the automatically generated LLR cluster label is ‘registered report’, the cluster label was manually replaced with ‘preregistration and registered reports’ to reflect the cluster content—which encompasses both preregistration and registered reports—more accurately.

The major citing articles by Cook *et al.* [99] and Noret *et al.* [98] both discuss recommendations for preregistration, where Cook *et al.* [99] also discusses the benefits of registered reports in special education. Other citing articles also discuss preregistration [101,102]. Similarly, the cited references include preregistered studies [80] and discussions on preregistration [103] and registered reports [104,105]. Preregistration involves the planning and documentation of research hypotheses and questions, intended procedures and materials, and data analysis plans [106]. Through pre-registration, analyses and outcomes resulting from predictions can be distinguished from analyses that are conducted after data collection, preventing QRPs such as *p*-hacking. Similarly, registered reports also involve planning and delineating research questions and methods before embarking on the research study. From there, registered reports move beyond preregistration where these research plans are submitted for peer review and form the primary basis for acceptance for publication. Adherence to the research plans means that researchers are unable to engage in QRPs such as employing various analytic approaches to reach statistical significance or selectively report positive results. Essentially, such a procedure discourages or even eradicates the need for QRPs in order to produce ‘positive’ results to meet the perceived publication bias. As stated in the major citing article by Götz *et al.* [100], preregistration and registered reports are potent avenues for a more realistic portrayal and understanding of (small) effect sizes in psychological science, which moves away from the publication bias for statistically significant results centred on expectations of large effect sizes. Hence, this cluster appears to suggest a relatively significant interest in preregistration and registered reports as more ‘popular’ open science practices being encouraged across different fields in the scientific community.

### 4.10. Additional considerations in questionable research practises research

Although this review has highlighted a number of key themes in the research on QRPs, there are a number of considerations regarding this research. Firstly, this study adopted a broad perspective in examining QRPs across scientific fields. It is important to note, however, that there does not appear to be a single definition as to what constitutes a QRP. In reality, there is evidence indicating varying perceptions and practise of QRPs in different scientific disciplines [107]. Notably, many QRPs were reported to only be found within one particular research area and the QRPs also appeared to be reported in different phases of the research process, where such differences in the perception of QRPs appears to stem from differences in disciplinary practises. For example, more problems in idea generation and design were reported in the social sciences sample as opposed to a majority of QRPs being reported in the analysis and reporting phases by the medical sciences sample in the study by Ravn & Sørensen [107]. Hence, these studies suggest that such differences across scientific fields should be taken into consideration when making recommendations for best practises in research and research integrity rather than implementing a blanket standard operating procedure.

Secondly, in line with the idea of varying perceptions of QRPs and the need to consider the nature of research across scientific disciplines, a perspective with greater nuance to the discussion on QRPs has also begun to emerge. The presumption of QRPs as problematic seems to underlie the majority of existing work in their approach towards QRP research. The need to consider the applicability of current recommended best practises across different methods within scientific fields as well as across scientific fields is highlighted by Rubin [108], where aspects of the ‘scientific method’ may not be directly applicable to each and every science, and a rigid implementation of recommended best practises across the board may not be practical. For example, recommendations targeting quantitative methods in psychology may not necessarily be entirely applicable to qualitative or mixed methods studies, where Reischer & Cowan [109] suggested recommendations for mixed methods studies in psychology in relation to existing open science practises. One such example is the relevance of replication studies where Reischer & Cowan [109] highlighted that direct replication may not be entirely practical for mixed methods studies and reiterated the need for multiple standards for quality science in terms of validity.

### 4.11. Limitations

There are a number of limitations to the scientometric approach employed in this study. First of all, the sample of documents used in the current analysis may not be exhaustive as it is limited to articles that are indexed by Scopus, meaning that there may be articles that were not included in the current analysis. In addition, the DCA is a quantitative analysis based on the number of citations and co-citations in the dataset. In doing so, DCA does not take into account qualitative aspects of the co-citation patterns. For this reason, in the current work, the scientometric analysis was followed by a qualitative discussion of the clusters [23,110]. The purely quantitative nature of the DCA analysis also means that the results may be influenced by inherent patterns of citations in the articles included for the analysis, such as citation bias, which refers to the preferential citation of research consistent with one’s own findings. [111] For example, Horbach *et al.* [112] have reported findings suggesting trends in the citing behaviour of the study by John *et al.* [2], where the nature of the citations of the study appeared to be increasingly superficial and less substantive over time. It should also be noted that since the formation of the clusters was based on the analysis of the dataset and not defined *a priori*, the analysis was exploratory in nature. Finally, the current study had not been preregistered and future scientometric studies can preregister the procedures involved before commencing with the data collection.

## 5. Conclusion

Research integrity and the prevalence of QRPs in individual fields of science have received increasing research attention over the years and the research has grown beyond just characterizing the severity of the prevalence of QRPs, where the scientometric results indicate thematic shifts in the research over time. Moreover, the review has highlighted that research into QRPs is concerned with the credibility and replicability of scientific research, where the research has gradually shifted its focus on more overt and ‘serious’ cases of research misconduct towards QRPs and their consequences on the replicability and credibility of research. More recently, the research on QRPS has moved to the efficacy of open science practises to counter the prevalence of engagement in QRPs by researchers. From the formation of clusters, it seems that preregistration and registered reports may be receiving the most research interest, whereas other open science practises such as the impact of data sharing and methods sharing is less understood. Nevertheless, the shift towards open science practises in research and their gradual uptake across different fields of science is an encouraging sign of the move towards quality research. As the open science movement continues to grow, more research into the application of these practises in science on the ground and their effect on scientific findings should be monitored and expanded to build and maintain a consistent quality of research.

## Data Availability

The dataset and script for the current work have been uploaded to Dryad in the following link: https://doi.org/10.5061/dryad.2fqz612tx [113].
